# COPD patients need more information about self-management: a cross-sectional study in Swedish primary care

**DOI:** 10.1080/02813432.2019.1684015

**Published:** 2019-11-07

**Authors:** Hanna Sandelowsky, Ingvar Krakau, Sonja Modin, Björn Ställberg, Anna Nager

**Affiliations:** a Academic Primary Health Care Centre, Stockholm County Council, Stockholm, Sweden;; b Section for Family Medicine and Primary Care, NVS, Karolinska Institutet, Stockholm, Sweden;; c Department of Medicine, Division of Clinical Epidemiology, Karolinska Institutet, Stockholm, Sweden;; d Department of Public Health and Caring Sciences, Family Medicine and Preventive Medicine, Uppsala University, Uppsala, Sweden

**Keywords:** Primary care, COPD, self-management, disease information needs, patient education, general practitioners, cluster randomized controlled trial

## Abstract

**Objective:** In Sweden, patients with chronic conditions, such as chronic obstructive pulmonary disease (COPD), often receive education at specialized nurse-led clinics at primary health care centers (PHCCs). Identifying patients’ needs for information about COPD is the key to individualized care. This study aimed to assess self-reported needs for information about COPD in primary care patients with either moderate (GOLD 2) or severe (GOLD 3) COPD and identify patient characteristics and exacerbation patterns associated with the findings.

**Design:** A cross-sectional study.

**Setting:** Twenty-four PHCCs in Stockholm, Sweden.

**Subjects:** Randomly selected primary care patients with COPD in GOLD stages 2 and 3 (*n* = 542).

**Main outcome measures:** The Lung Information Needs Questionnaire (LINQ) was used to assess perceived information needs. Spirometry results and descriptive, self-reported data on patient factors such as exacerbation history, treatment, smoking, weight/height, comorbidities, health care contacts, education and symptoms were collected.

**Results:** Overall, the greatest reported needs were for information about self-management and diet. GOLD 2 patients (68%) expressed greater needs for information than GOLD 3 patients (32%). We found significant associations between high information needs and patient-related factors such as ‘No assigned GP’ (OR = 4.32 [95% CI 2.65–7.05]) and ‘No contact with COPD nurse in the past 12 months’ (OR = 1.83 [95% CI 1.19–2.81]).

**Conclusion:** COPD patients felt they knew too little about self-management of their disease. Low information needs were strongly associated with continuity in patient-GP consultations and moderately associated with contact with a COPD nurse. These associations were strongest in patients with moderate COPD.

**Key points:** As patients with COPD often have multimorbidity, identifying patients’ needs for information about COPD is essential to providing individualized patient education and care. In this study of 542 patients from 24 Swedish primary care centers, we found that:

- Patients with COPD, particularly those with moderate airflow limitation (i.e. GOLD 2) felt they needed more information about COPD than currently provided by health care professionals in primary care.
- Low information needs were strongly associated with continuity in patient-GP consultations and moderately associated with contact with a COPD nurse. GPs’ part in COPD patient education should not be overlooked, as individualized COPD care relies on GPs’ expertise in managing patients with multimorbidity.

## Introduction

Chronic obstructive pulmonary disease (COPD) is one of the leading causes of morbidity and mortality worldwide [1]. Smoking cessation and prevention of acute exacerbations are the two most important COPD treatment actions associated with improved prognosis [2]. In addition to inhaled medications, exercise and dietary treatments are essential for reducing patient’s symptoms. Comorbidities are common and make patient education complex, worsen health status and prognosis, and increase healthcare costs [2–4].

Patients’ self-management skills are crucial for optimal and individualized care of complex, long-term conditions such as COPD. Earlier research indicates that participating in nurse-led self-management programs improves self-efficacy, health-related quality of life, exercise capacity, and levels of anxiety [5]. Patients also have fewer unscheduled physician visits, hospital admissions, and days in the hospital [6]. Patient education by nurses has thus become an essential component in guidelines for optimal, interprofessional COPD care in Sweden [7]. The growing number of specialized COPD nurses in primary care offers relief to general practitioners (GPs), who are often too few, short of time, and therefore offer suboptimal continuity of care. Moreover, COPD nurses’ special skills are often important, as many GPs have a low level of knowledge about COPD [8]. However, focusing on nurses may result in GPs deprioritizing their own patient education efforts.

The Global Initiative for Chronic Obstructive Lung Disease (GOLD) has defined and revised COPD severity grades to help clinicians assess the severity and prognosis of the disease and determine how to manage the patient. Whereas the older GOLD 1–4 classification system (2013) used a patient’s airflow limitation as the basis of disease management recommendations [9], symptom burden and exacerbation history are the basis of the newer GOLD A–D classification system (2017) [10]. Although use of the GOLD A–D system is underway, GPs in Sweden are still more familiar with the GOLD 1–4 system, not least because GOLD 1–4 is still included in the official guidelines in Sweden [11].

Studies about the views and knowledge that COPD patients in primary care have about their disease are fairly scarce. Our study aimed to reduce an existing research gap by assessing self-reported needs for information about COPD in primary care patients with either moderate (GOLD 2) or severe (GOLD 3) COPD. In addition, we aimed to examine the association between these needs, exacerbation history and other patient characteristics.

## Material and methods

Participants in this cross-sectional study were patients from 24 primary health care centers (PHCCs) in Stockholm County. The study took place between September 2014 and May 2015. The 24 PHCCs participated in a cluster randomized controlled trial (the PRIMAIR Study). PRIMAIR aimed to reduce the existing gap between guidelines and practice by improving the quality of COPD management by GPs. It studied the effects of continuing medical education in COPD for GPs and included both GP and patient outcomes. A detailed description of the methodology and interventions of PRIMAIR is found in the previously published study protocol [12]. As the current article presents the results of a cross-sectional study that used baseline patient outcome data from PRIMAIR, it has been written in accordance with the Strengthening the Reporting of Observational Studies in Epidemiology (STROBE) statement: guidelines for reporting observational studies [13] (Supplementary file 1).

Eligible patients had a spirometry-confirmed ICD-10 diagnosis of COPD (J44.0–J44.9), GOLD 2 or 3 (moderate or severe COPD, i.e. forced expiratory volume of one second [FEV1], 30–79% of predicted). A total of 957 randomly selected primary care patients, 40–45 per PHCC, were invited to participate in the study. Patients who agreed to participate replied to the invitation by providing written informed consent and completing a questionnaire [12] that consisted of questions about exacerbation history, treatment, smoking, weight/height, comorbidities, health care contacts and education. In addition, the validated Lung Information Needs Questionnaire (LINQ, http://www.linq.org.uk/) [14] was included to assess patients’ perceived needs for information about COPD. The LINQ is primarily used as a tool to help professionals plan and provide individualized patient education. It covers six domains: ‘Disease knowledge’, ‘Medication’, ‘Self-management’, ‘Smoking’, ‘Exercise’ and ‘Diet’. The minimum score per domain was 0, and the maximum varied between 2 and 6, depending on the domain. The maximum possible total score was 25. The higher the score, the greater the patient’s perceived needs for information about COPD. The minimal clinically important difference (MCID) in LINQ scores is 1 point. PRIMAIR included three other validated questionnaires. The Clinical COPD Questionnaire (CCQ) [15] and the COPD Assessment Test (CAT) assessed the impact of COPD symptoms on health status [16], and the Modified Medical Research Council dyspnea scale (mMRC) [17] graded the impact of breathlessness on daily activities. Lung function measures, including FEV1 and forced vital capacity (FVC), age, and gender were collected from patients’ medical records.

A COPD exacerbation was defined as a patient-reported intermittent period of deterioration in the disease in the previous 6 months that had warranted an unscheduled or emergency visit to a PHCC or hospital and/or additional medication with antibiotics and/or oral steroids.

### Statistical analysis

Summary statistics such as means, proportions, and measures of dispersion were computed using standard parametric methods. Further standard parametric analyses were conducted by gender, GOLD stage, and exacerbation history. Logistic regression was used to analyze variables associated with levels of information needs and also provided odds ratios and their 95% confidence intervals (CIs). After a preliminary analysis in a univariate model, variables with a *p* value <0.1 were entered into a multivariable model in which *p* values <0.05 indicated statistical significance. We then performed a binomial logistic regression analysis. For that, we defined a total LINQ score above the mean, i.e. ≥11 points as ‘high’ (i.e. high information needs) and a total score <11 points as ‘low’ (i.e. low information needs). The power calculation was based on the mean and standard deviation of the CCQ and the minimal clinically important difference of 0.44 in the CCQ [15,18], as CCQ was the main outcome measure of the PRIMAIR Study. According to the power calculation, 460 patients were required. The statistical analysis was performed using SPSS software (IBM SPSS Statistics for Windows, Version 25.0, Released 2013, IBM Corp, Armonk, NY).

## Results

A total of 542 of the invited patients (57%) responded. The non-response rate (*n* = 415, 43%) was independent of gender, but non-responders were slightly but significantly younger than responders (70.3 vs. 72.0 years, *p* = 0.01) and more often in GOLD 2 than in GOLD 3 (68% vs. 57%, *p* = 0.001). Other characteristics of patients who did not respond were not recorded.

### Patient characteristics

Table 1 shows the *overall patient characteristics* and a comparison of characteristics of *patients with moderate (GOLD 2) and patients with severe COPD (GOLD 3)*.

**Table 1.** Descriptive data on patients in total (*n* = 542) and by GOLD stage, and a comparison of the GOLD 2 and GOLD 3 patients.

Characteristics n (%)	All patients *n* = 542 (100.0)	GOLD 2^a^*n* = 370 (68.3)	GOLD 3 *n* = 172 (31.7)	*p* Value
Age				
Years, mean [95%CI]	72.0 [71.3–72.7]	71.3 [70.4–72.2]	73. 6 [72.3–74.8]	0.005
Age distribution, n (%)				
Age 35–64 years	94 (17.3)	77 (20.8)	17 (9.9)	
Age 65–79 years	334 (61.6)	221 (59.7)	113 (65.7)	
Age 80–93 years	114 (21.0)	72 (19.5)	42 (24.4)	
Exacerbation				
Acute exacerbation in the last 6 months	183 (33.8)	104 (28.1)	79 (45.9)	<0.001
Gender				
Female, n (%)	316 (58.3)	220 (59.5)	96 (55.8)	n.s.
Education				
>9 years, n (%)	260 (45.2) (Missing data = 27)	192 (49.0)	68 (37.3)	0.013
Care provider contact^b^				
General practitioner	389 (72.2) (Missing data = 3)	260 (70.7)	129 (75.4)	n.s.
COPD nurse	191 (36.8)	120 (32.1)	71 (41.3)	0.047
Physiotherapist	71 (13.5) (Missing data = 2)	37 (10.4)	34 (19.9)	0.002
Nutritionist	37 (7.2) (Missing data = 1)	17 (4.8)	20 (12.3)	0.003
Occupational therapist	20 (3.8) (Missing data = 15)	11 (3.1)	9 (5.3)	0.033
Social worker	6 (1.1) (Missing data = 15)	2 (0.6)	4 (2.3)	n.s.
Smoking intensity				
Pack years^c^, mean [95%CI]	32.9 [31.2–34.7] (Missing data = 74)	32.6 [30.5–34.7]	33.7 [30.4–37.0]	n.s.
Comorbidity				
Hypertension, n (%)	267 (49.3)	186 (50.3)	81 (47.1)	n.s.
Asthma, n (%)	155 (28.9)	105 (28.7)	50 (29.2)	n.s.
Heart disease, n (%)	120 (22.1)	76 (20.5)	44 (25.6)	n.s.
GERD^d^, n (%)	108 (19.9)	76 (20.5)	32 (18.6)	n.s.
Anxiety/depression, n (%)	99 (18.3)	74 (20.0)	25 (14.5)	n.s.
Type 2 diabetes, n (%)	70 (12.9)	45 (12.2)	25 (14.5)	n.s.
Chronic pain, n (%)	68 (12.6) (Missing data = 3)	47 (12.8)	21 (12.3)	n.s.
No comorbidity, n (%)	88 (16.2)	62 (16.8)	26 (15.1)	n.s.
FEV1^e^ after bronchodilator				
% of predicted value	56.4 [55.2–57.5]	64.0 [63.2–64.9]	40.1 [39.2–41.0]	<0.001
Body mass index				
Kg/m^2^ [95%CI]	25.6 [25.2–26.1] (Missing data = 32)	25.6 [25.4–26.5]	24.9 [24.1–25.8]	0.038
Medication				
No regular inhaled medication, n (%)	70 (12.9)	62 (16.8)	8 (4.7)	<0.001
ICS only, n (%)	19 (2.9)	14 (3.8)	5 (2.9)	n.s.
LAMA and/or LABA only, n (%)	134 (24.7)	100 (27.0)	34 (19.8)	n.s.
ICS + LAMA only, or ICS + LABA only, n (%)	99 (18.3)	72 (19.5)	27 (15.7)	n.s.
ICS + LABA + LAMA only, n (%)	218 (40.2)	120 (32.4)	98 (57.0)	<0.001
Health status^f^				
CAT, total score of 0–40, mean [95%CI]	15.2 [14.5–15.9] (Missing data = 30)	14.0 [13.2–14.9]	17.8 [16.5–18.9]	<0.001
CCQ, mean score of 0–6, mean [95%CI]	1.89 [1.79–1.99] (Missing data = 13)	1.71 [1.59–1.83]	2.27 [2.08–2.45]	<0.001
mMRC dyspnea score ≥ 2 points, n (%)	222 (42) (Missing data = 14)	124 (55.9)	98 (44.1)	<0.001

CI: Confidence interval; OR: Odds ratio; LABA: Long acting beta agonist; SAMA: Short acting muscarine agonist; LAMA: Long acting muscarine agonist; ICS: Inhaled corticosteroid.

^a^COPD severity grade according to the Global Initiative for Chronic Obstructive Lung Disease (GOLD): GOLD 2 (moderate, 50% ≤ FEV1 < 80% of predicted), GOLD 3 (severe, 30% ≤ FEV1 < 50% of predicted).

^b^Derived from question 27 in the patient questionnaire: ‘Where do you normally go to have your lung disease checked?’ (response alternatives: multiple choice) and questions 29 through 33: ‘Have you visited a/an [profession] on account of your lung disease in the past year?’ (response alternatives: ‘yes’ and ‘no’).

^c^Number of pack years = (number of cigarettes smoked per day × number of years smoked)/20.

^d^Gastroesophageal reflux disease.

^e^Forced expiratory volume of one second.

^f^The COPD Assessment Test (CAT), score 0–40; the Clinical COPD Questionnaire (CCQ), score 0–6; the mean of the minimum of 8 items of 10; the Modified Medical Research Council Dyspnea (MRC) scale, score 0–4.

A comparison of *patients who had exacerbated* (*n* = 183) *and patients who had not exacerbated* (*n* = 359) showed that those who had exacerbated had significantly higher symptom burden (CAT 19.1 [17.9–20.3] vs. 13.2 [12.4–14.0], CCQ 2.53 [2.35–2.71] vs. 1.56 [1.45–1.66], and mMRC scores of ≥2 points 58% vs. 34%, all *p* values < 0.01). They also had significantly more contacts with different care providers than their counterparts who had not exacerbated. There were no significant gender differences in exacerbation frequency by GOLD stage, but women who had exacerbated had smoked significantly less than men who had exacerbated.

In a comparison of *GOLD 2 and 3 patients who had exacerbated*, we found that GOLD 3 patients had worse disease-related health status (CCQ scores 2.67 [2.38–2.96] vs. 2.42 [2.19–2.65], *p* < 0.001), more often rated their dyspnea severe (mMRC scores of ≥ 2, 68% vs. 52%, *p* = 0.031), and had a lower body mass index (24.8 vs. 26.6, *p* = 0.045). They more often used ‘triple therapy’ (i.e. a combination of long-acting beta agonist [LABA], long-acting muscarine agonist [LAMA], and inhaled corticosteroids [ICS] (64.8% vs. 49.0%, *p* = 0.036). However, exacerbating GOLD 2 patients reported anxiety and/or depression more often than did exacerbating GOLD 3 patients (29% vs. 10%, *p* = 0.002).

### Patients’ information needs (LINQ)

The mean total LINQ score was 11 (11.03 [95% CI 10.67–11.39]) of the maximum 25 points (44.1% of the maximum total LINQ score.) Overall, GOLD 2 patients reported greater needs for information about COPD than GOLD 3 patients (total LINQ score 11.40 vs. 10.23, *p* = 0.003. Although the difference was small, it exceeded the MCID of 1 point and should thus be considered clinically significant.

**Figure 1.**
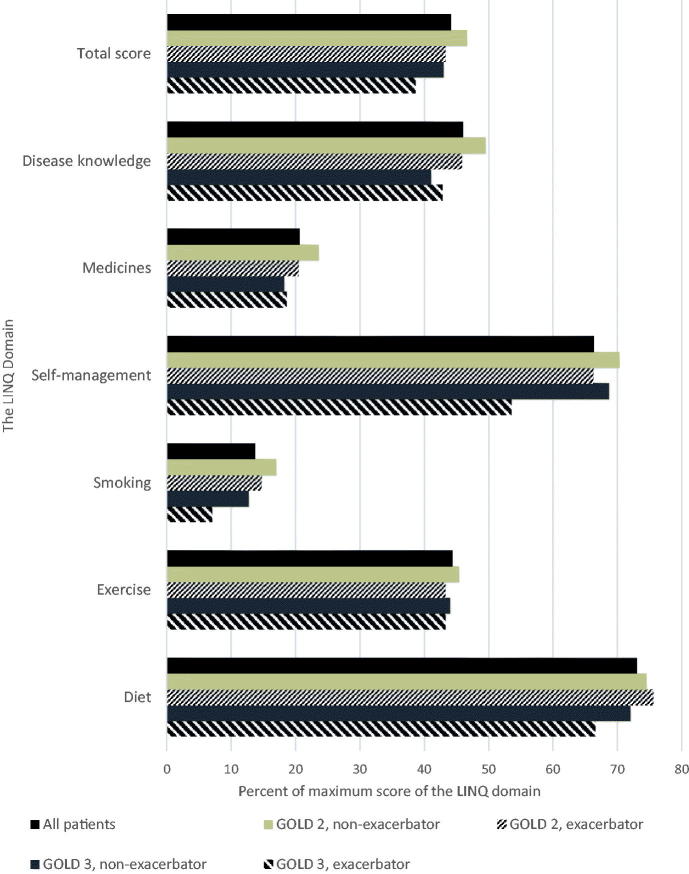
Results of The Lung Information Needs Questionnaire (LINQ), mean total score and mean score for each domain presented as percentage of maximum total score and percentage of maximum scores for each of the six domains. Maximum total score, 25 points; scores in each domain vary from 0–2 to 0–5 points.

Figure 1 shows the overall results for each LINQ domain. Generally, patients expressed a great need for information in the domains ‘Diet’ and ‘Self-management’ and a moderate need for information about ‘Disease knowledge’ and ‘Exercise’. They reported the least need for information about ‘Medicines’ and ‘Smoking’. However, complementary questions in the questionnaire revealed that almost 60% who were current smokers or had quit smoking in the last five years (*n* = 382) had not been *offered* smoking cessation support, and 80% had not previously been *given* such support by their GPs or nurses. GOLD 2 patients who had exacerbated reported significantly higher needs for information about ‘Self-management’ and ‘Smoking’ than their GOLD 3 counterparts. Patients with moderate disease reported significantly higher needs for information than patients with severe disease in all domains except ‘Exercise’ and ‘Diet’, in which scores were similar in both groups.

Table 2 shows the results of a binomial logistic regression analysis, performed for the whole study population, with the LINQ score of ≥11 points as the response variable. Not having an assigned GP was strongly associated with high perceived information needs. Further analysis showed that ‘no assigned GP’ and ‘current smoking’ were positively associated with high information needs, particularly in GOLD 2 patients.

**Table 2.** Patient-related factors associated with high perceived needs for information^a^ in the study population.

Factor	Unadjusted OR^b^	95% CI for unadjusted OR	*p* Value	Adjusted OR^c^	95% CI for adjusted OR	*p* Value
No assigned GP	4.16	2.62–6.61	<0.001	4.32	2.65–7.05	<0.001
No contact with COPD nurse in the past 12 months	1.94	1.31–2.88	0.001	1.83	1.19–2.81	0.006
More than nine years of formal education	1.41	0.96–2.08	0.076	1.62	1.07–2.46	0.024
Current smoker	1.72	1.14–2.60	0.010	1.60	1.02–2.52	0.043
No exacerbations in the past 6 months	1.44	0.98–2.14	0.066	1.59	1.03–2.46	0.037

CI: confidence interval; OR: odds ratio.

^a^Odds ratios for scoring above the study population’s mean total LINQ score of ≥11 (missing LINQ data, *n* = 123).

^b^Unadjusted logic regression analysis.

^c^Logistic regression analysis with all factors adjusted for each other.

## Discussion

### Statement of principal findings

This Swedish study of the information needs of COPD patients had three main findings. First, the results of the LINQ made us suspect that the majority of patients have a substantial need for information about COPD. Patients expressed particular insecurity about self-management and diet. Second, although patients with moderate COPD were generally in better medical condition than patients with severe COPD, they reported greater needs for information about the disease. Third, high information needs were strongly associated with discontinuity in GP care and moderately associated with having had no contact with a COPD nurse in the past year. This association was particularly strong in patients with moderate COPD.

### Strengths and weaknesses

Self-reported data, especially from the validated questionnaires used in this study (LINQ, CCQ, CAT and mMRC), are valuable because they provide a more nuanced picture than the information registered in medical records. An estimated 40–50% of exacerbations are not reported to health care professionals [19], so first-hand information from patients is valuable. However, self-reported data can be misleading, as patients may differ in understanding and remembering. Another limitation was the inability to distinguish between ‘never-smokers’ and ‘former smokers’ in the LINQ. However, the number of never-smokers was probably very small.

As with all questionnaires, patients’ intuitive, subjective sense of their information needs may differ from the information found in their actual responses to the LINQ. Combining the LINQ with a questionnaire that assessed patients’ levels of knowledge about COPD could have provided more detailed information useful in individualizing patient education [20]. However, the brevity of the LINQ was a strength, as PRIMAIR included nine pages of questionnaires. It also gave us the opportunity to contribute new information about the LINQ, which has been validated but is relatively unstudied. We did not specifically examine the feasibility of using the LINQ as part of a patient-professional consultation. However, a Canadian research group recently found that the LINQ was useful even in brief consultations [21].

A limitation in our analysis of the LINQ data was the lack of a validated cut-off level for the total score. To improve our understanding about which levels of perceived information needs we should categorize as ‘high’ and ‘low’, we performed different types of regression analyses. The results of these analyses did not differ substantially. We therefore chose to present the results of the binomial analysis because of its relative simplicity. However, in the absence of a validated cut-off level for the total score, we suggest that the results can only be generalized to other primary care contexts with caution. In the future, researchers should consider validating a cut-off between high and low levels of perceived information needs as measured by LINQ.

### Findings in relation to other studies

#### Results of the LINQ

The mean total LINQ score indicated that all patients, but particularly those with moderate COPD, feel they need more information about the disease and thus more support and education from health care professionals than they currently receive. In a 2008 study of a rehabilitation intervention, LINQ findings were similar to ours, which suggests that it is difficult to achieve optimal patient education [14].

The domain ‘Self-management’ consisted of questions about whether the patient was confident about assessing and managing an acute exacerbation. The high scores in this domain may reflect the overall insecurity and fear of deteriorations that are typical for COPD patients [22]. ‘Diet’ was another LINQ domain that yielded high scores, which probably reflects the previously reported relatively low status of dietary treatments in the management of COPD [23]. Unlike in the rehabilitation study [14], patients in our study did not express high information needs in the ‘exercise’ domain. We reason that this is a consequence of the generally improved awareness of the effects of exercise in COPD care [24].

We were not surprised to find ‘smoking’ and ‘medicines’ among the areas in which the patients felt they needed the least additional information. Most health care professionals are well aware of these subjects, regard them as important in COPD care [25,26], and thus seem likely to keep their patients relatively well-informed about them. However, the LINQ questions about smoking were only targeted at current smokers, which may have skewed the outcome in a positive direction. It is difficult to quit smoking without motivational and pharmacological support [27]. Earlier research shows that doctors rarely take active measures to help patients quit smoking [28]. Patients may also have demonstrated a false sense of confidence when assessing their needs for information about medicines, as studies consistently report that many patients do not use their inhaler devices correctly and are unaware of it [29].

#### Educating COPD patients

Quitting smoking at an early stage of the disease is the best way to reduce COPD progression, morbidity, and mortality [30]. The large number of patients with moderate COPD in our study population who were current smokers indicates that early patient education and smoking cessation support are needed. Previous research shows that written action plans; health coaching with motivational interviewing or via internet-based tools; and patient factors such as younger age, better lung function measures, absence of cardiac morbidity, and not living alone may improve the outcome of patient education and self-management support and counteract COPD exacerbations [31–33].

Patient-nurse consultations may be particularly important when issues like fear, self-blame, ambivalence toward treatment and lack of personal empowerment dominate the consultation, as these issues reduce the odds of positive outcomes [34,35]. In primary care in Sweden, COPD nurses play a central role in patient education [6,36]. In our study population, about a third of all patients, regardless of GOLD stage, had met a COPD nurse in the previous 12 months.

In general, we observed both a relative lack of care for patients with moderate disease and potential consequences of this inattention. Patients with moderate COPD who had exacerbated had less medication than their counterparts with severe COPD despite an equal symptom burden. In general, less of the care patients with moderate COPD received was interprofessional [24]. Patients with moderate COPD also reported greater needs for information and support than patients with severe COPD. A likely explanation is that the onset of education for patients with moderate COPD often occurs late – only when they start to experience acute exacerbations. There may also be a link between this late onset of education and the more frequent anxiety and/or depression experienced by patients with moderate COPD who had exacerbated than their counterparts with severe disease. A low level of knowledge about COPD is a significant risk factor for anxiety and depression in COPD patients [37].

COPD is a chronic disease that especially affects people who may already be vulnerable. This includes those with low socioeconomic status and a low level of education, who often have coexisting risk factors for COPD, such as heavy smoking and multimorbidity [38]. Many of the participants in the study had a low level of formal education. Previous research indicates that low levels of education reduce patients’ chances to benefit from patient education [39]. The positive association between a higher level of education and greater information needs in our study population (especially in GOLD 2 patients) may indicate that highly educated people, in general, have a more pronounced subjective desire for information. Whether low socioeconomic status contributes to patients’ tendency to participate in patient education or to care providers’ tendency to offer patients interprofessional COPD care is a question for further study.

Our results highlight the importance of well-functioning patient-GP relationships. Low needs for information about COPD were strongly associated with continuity in patient-GP consultations. However, whether or not decreased needs for such information are associated with improved health outcomes is a question for further research. Although heavy workloads and staff shortages may make GPs prioritize other tasks over education for COPD patients, GPs’ role in patient education should not be overlooked. Our results suggest that GPs should actively participate in educating patients rather than solely rely on other professionals. Patient education by GPs should be based on well-functioning communication between the GP and the patient and thus on a mutual understanding of a need for a partnership in care [40]. Education should start when the GP informs the patient about the diagnosis, as early education is particularly important to help patients understand and learn to manage their disease [22]. As many of primary care patients with comorbidity and/or multimorbidity only have care contacts with GPs, rather than interprofessional care teams [41], GPs’ competences in providing holistic care and patient education are specifically needed. Multimorbidity is common in patients with COPD [4]: 84% in our study population. This, in theory, could give GPs many opportunities to educate patients. Paradoxically, because of time constraints, insufficient interprofessional cooperation, and their own negative views of COPD, Swedish GPs seem to deprioritize management of COPD in patients with multimorbidity [25]. To reduce the risk of deprioritizing COPD, GPs’ continuing medical education should aim to improve GPs’ level of knowledge of evidence-based COPD care [26]. Furthermore, as the feasibility of disease-specific guidelines for patients with complex needs is limited [41], continuing medical education should aim to improve GPs’ holistic management of patients with multimorbidity and to clarify the role and tasks of GPs in an interprofessional team.

## Conclusion and implications

COPD patients felt they needed more information about self-management. Continuity in patient-GP relationships (having an assigned GP), was strongly associated with low information needs. The results thus highlight the importance of GPs’ role in COPD care and patient education even at an early stage of COPD. We believe this real-life study can help motivate health care professionals and their educators to take further actions to improve COPD care.

## Supplementary Material

Supplemental Material
